# Supplementary material for: Activation of DR3 signaling causes loss of ILC3s and exacerbates intestinal inflammation
Source: Nat Commun. 2019 Jul 29;10:3371. doi: 10.1038/s41467-019-11304-8 (PMC6662828; doi:10.1038/s41467-019-11304-8)
Supplement: Supplementary file 1 — Supplementary Information [file 41467_2019_11304_MOESM1_ESM.pdf]

Activation of DR3 signaling causes loss of ILC3s and exacerbates intestinal inflammation

Li, Shi, et al.

Supplementary Figure 1

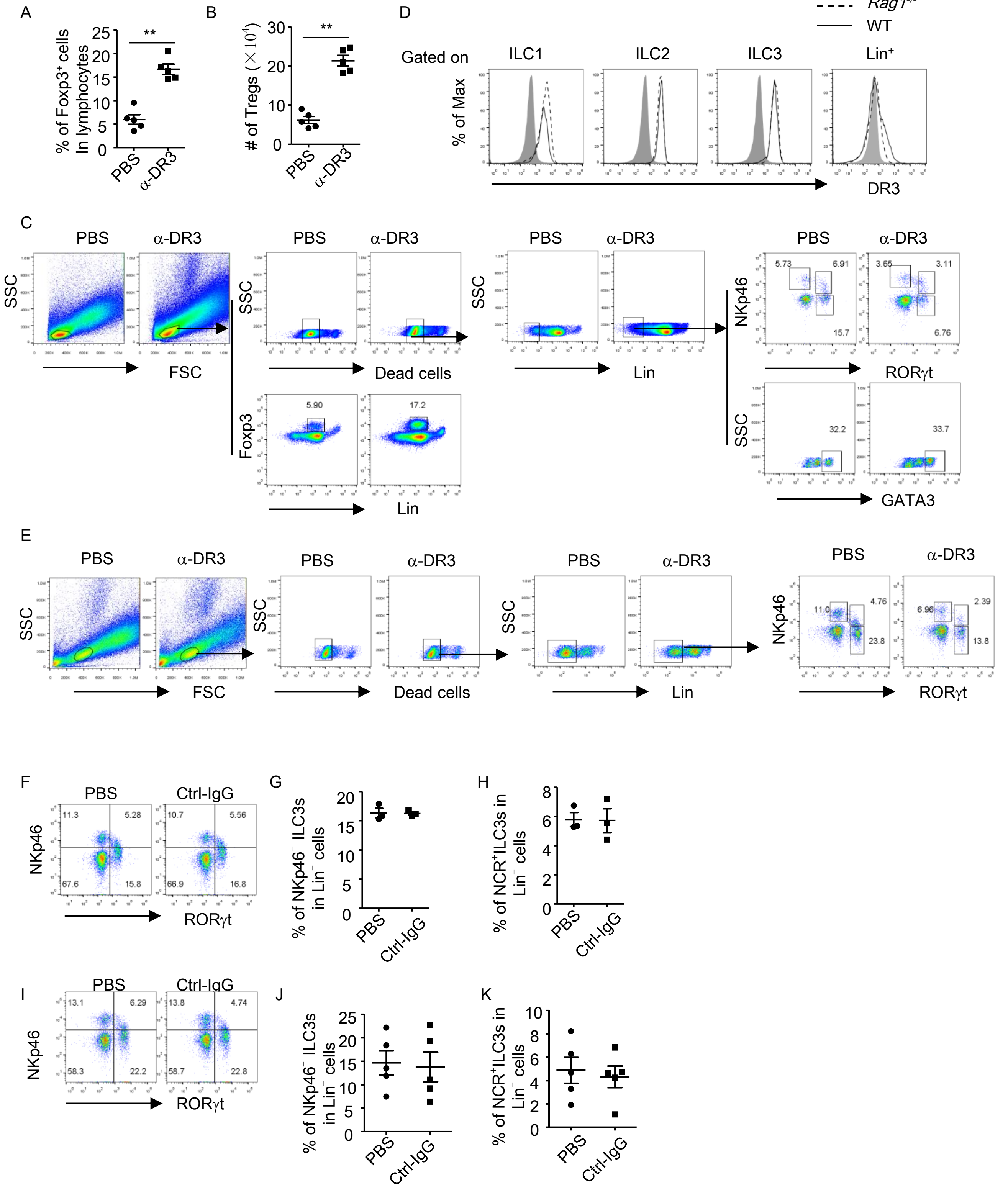

Supplementary Figure 1. DR3 is expressed by intestinal ILCs and α-DR3 expands large intestinal Tregs.

(A and B)Wild-type mice were treated with α-DR3 antibody (4C12) and large intestinal lamina propria lymphocytes (LPLs) were isolated for analysis 4 days later. (A) Expression of Lineage (Lin, CD3, B220, CD11b, CD11c) markers and Foxp3 was analyzed by flow cytometry. Percentages of $Lin^{+}Foxp3^{+}$ (Tregs) cells gated on lymphocytes were shown. (B) Total numbers of Tregs were shown. (C) Gating strategy for Figures 1A-1E. (D) Histogram expression of DR3 gated on ILC1 ($Lin^{-}NKp46^{+}ROR\gamma t^{-}$), ILC2 ($Lin^{-}GATA3^{high}$)， ILC3 ($Lin^{-}ROR\gamma t^{+}$) and Lin^{+} cells ($CD11b^{+}CD11c^{+}$ for *Rag1*$^{-/-}$ mice and $CD3^{+}B220^{+}CD11b^{+}CD11c^{+}$ for wild-type mice) from large intestinal LPLs of wild-type (solid line) or *Rag1*$^{-/-}$ mice (dashed line) under the steady-state was analyzed by flow cytometry. Shaded area was fluorescence minus one (FMO) staining from *Rag1*$^{-/-}$ mice as negative control. (E) Gating strategy for Figures 1G-1I. (F-H) Wild-type mice or *Rag1*$^{-/-}$ mice (I-K) were treated with PBS or Hamster IgG. Large intestinal LPLs were isolated 4 days later. (F-K) Expression of RORγt, NKp46 and lineage markers was analyzed by flow cytometry. (F and I) Expression of RORγt and NKp46 gated on Lin^{-} cells were shown. (G，H，J and K) Percentages of $NKp46^{-}$ILC3s ($Lin^{-}NKp46^{-}ROR\gamma t^{+}$) and NCR^{+}ILC3s ($Lin^{-}NKp46^{+}ROR\gamma t^{+}$) gated on Lin^{-} cells were shown. Data are means ± SEM. (A-K) Data are representative of at least two independent experiments. Source data are provided as a Source Data File.

Supplementary Figure 2

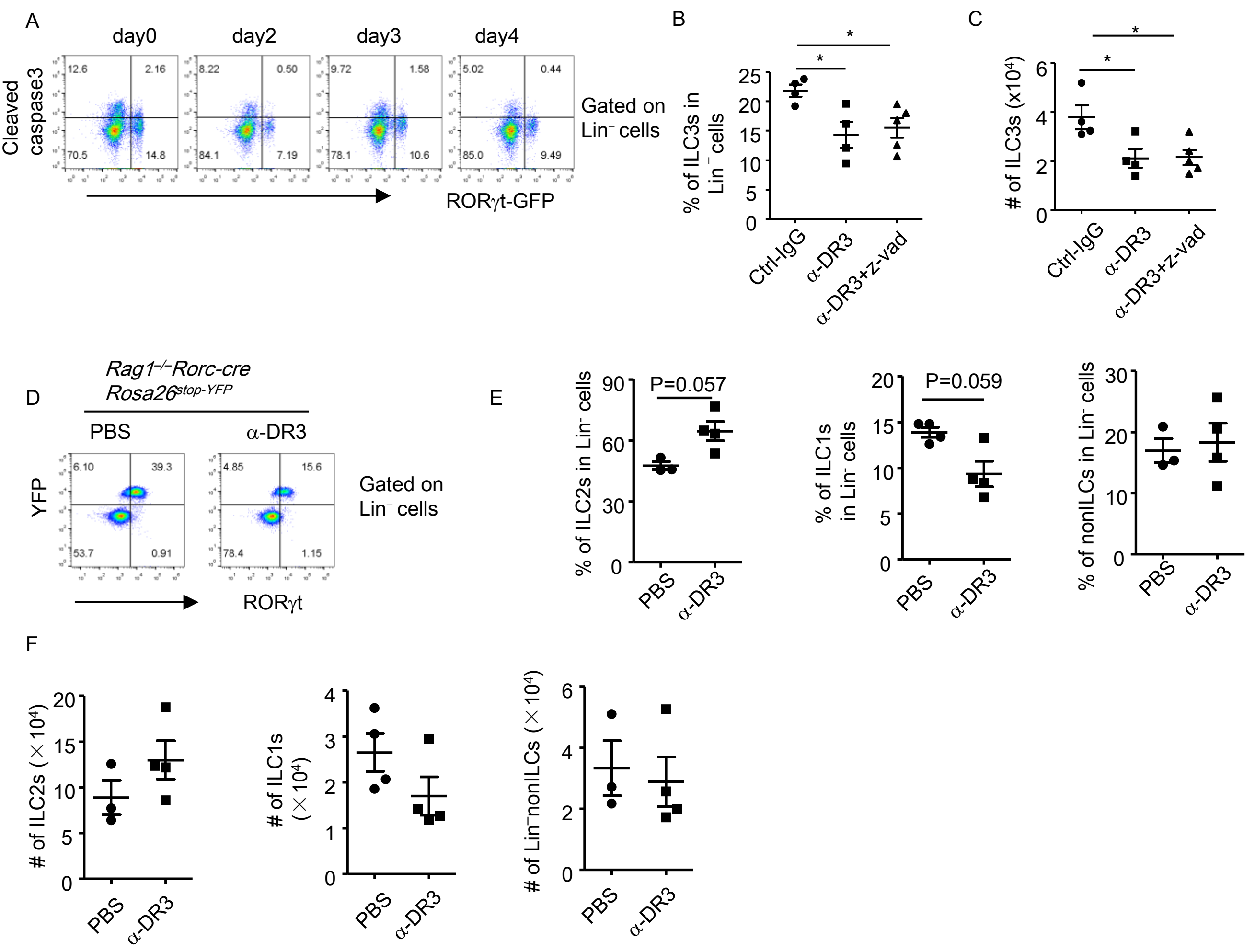

Supplementary Figure 2. Loss of ILC3s driven by α-DR3 is not due to cell apoptosis or fate conversion *in situ*.

Rorc$^{gfp/+}$ (A), *Rag1*$^{-/-}$*Rorc-creRosa26*$^{stop\text{-}YFP}$ (D) or *Rag1*$^{-/-}$ mice (E and F) were treated with α-DR3. Large intestinal LPLs were analyzed by flow cytometry 4 days later (B-F) or at indicated time points upon α-DR3 treatment (A). (A-F) Expression of Lin, RORγt-GFP, RORγt (intracellular staining), cleaved caspase 3, YFP, GATA3 and NKp46was analyzed by flow cytometry. (A) Expression of cleaved caspase 3 and RORγt-GFP gated on Lin$^-$ cells were shown. (D) Expression of YFP and RORγt gated on Lin cells were shown. Percentages of ILC2 (Lin$^-$GATA3$^+$), ILC1 (Lin$^-$NKp46^{+-}RORγt$^-$) and nonILC (Lin$^-$GATA3$^-$NKp46$^-$RORγt$^-$) in Lin$^-$ cells (E) and total numbers of indicated populations (F) were shown. (B and C) Wild-type mice were treated with Hamster IgG or α-DR3. α-DR3-treated mice were treated daily with DMSO or z-VAD-FMK (300ug). Expression of Lineage markers, RORγt and NKp46 gated on Lin$^-$ cells from large intestinal LPLs were analyzed by flow cytometry on day 4 after α-DR3 treatment. (B) Percentages of ILC3s (Lin$^-$RORγt$^+$) gated on Lin$^-$ cells were shown. (C) Total numbers of ILC3s from indicated groups were shown. (B, C, E and F) Data are means ± SEM. (B and C) Statistical analyses were performed using Student's t-test. (A-F) Data are representative of at least 2 independent experiments. Source data are provided as a Source Data File.

Supplementary Figure 3

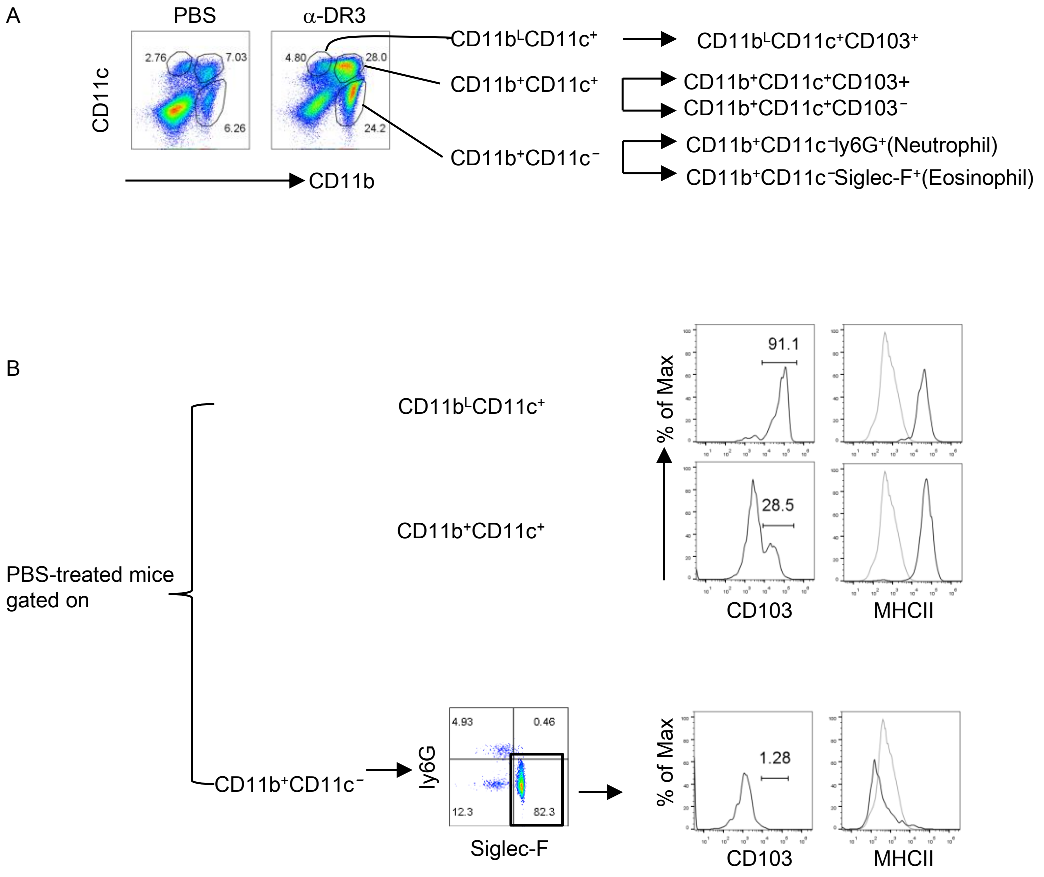

Supplementary Figure 3. Gating strategy for analysis of CD11b^{+} myeloid cells.
Rag1$^{-/-}$ mice were treated with PBS or α-DR3 antibody and large intestinal lamina propria lymphocytes (LPLs) were isolated for analysis 4 days later. (A) CD11b^{L}CD11c^{+} (CD11b^{L}CD11c^{+}CD103^{+}) cells, CD11b^{+}CD11c^{+}CD103^{+} cells, CD11b^{+}CD11c^{+}CD103^{-} cells, eosinophils (CD11b^{+}CD11c^{-}Siglec-F^{+}) and neutrophils (CD11b^{+}CD11c^{-}Ly6G^{+}) described in Figure 4 were identified accordingly. (B) The histograms (black solid lines) of expressions of MHCII and CD103 gated on subsets of CD11b^{low}(CD11b^{L}) and CD11b^{+} cells were shown. Light-black solid line was served as a negative gating for MHCII gated on CD11b^{-}CD11c^{-} cells. Data are representative of at least 2 independent experiments.

Supplementary Figure 4

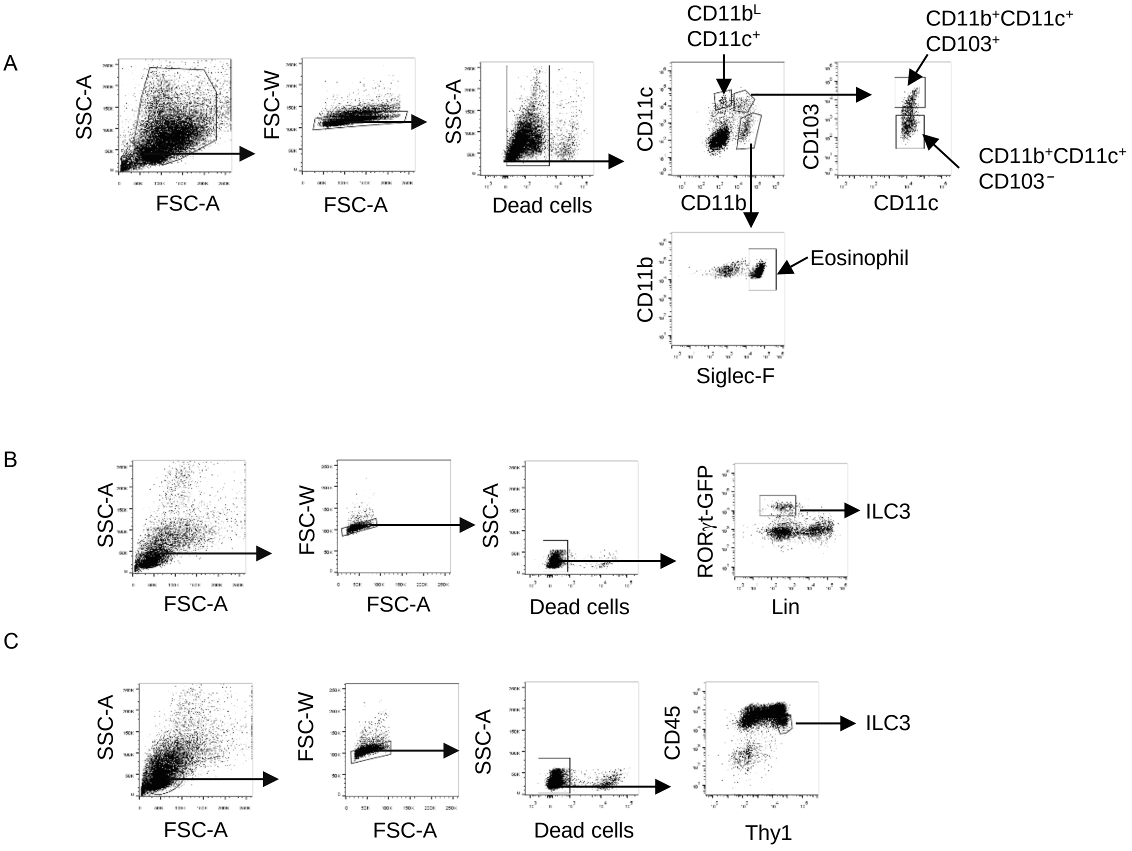

Supplementary Figure 4. Gating strategies used for cell sorting. (A) Gating strategy to sort CD11b^{L}CD11c^{+}, CD11b^{+}CD11c^{+}CD103^{+} cells, CD11b^{+}CD11c^{+}CD103^{-} cells and eosinophils (CD11b^{+}CD11c^{-}Siglec-F^{+} cells) from *Rag1*$^{-/-}$ mice treated with PBS or α-DR3 for experiments presented on Figure 4F and 4G. (B) Gating strategy to sort ILC3s from *Rag1*$^{-/-}$*Rorc*$^{gfp/+}$ mice treated with PBS or α-DR3 for experiments presented on Figure 5A, Supplementary Figures 5A-5B and Supplementary Data 1. (C) Gating strategy to sort ILC3s (Thy1.2^{high}CD45^{intermediate} cells) from *Rag1*$^{-/-}$ mice in experiments presented on Figure 5C.

Supplementary Figure 5

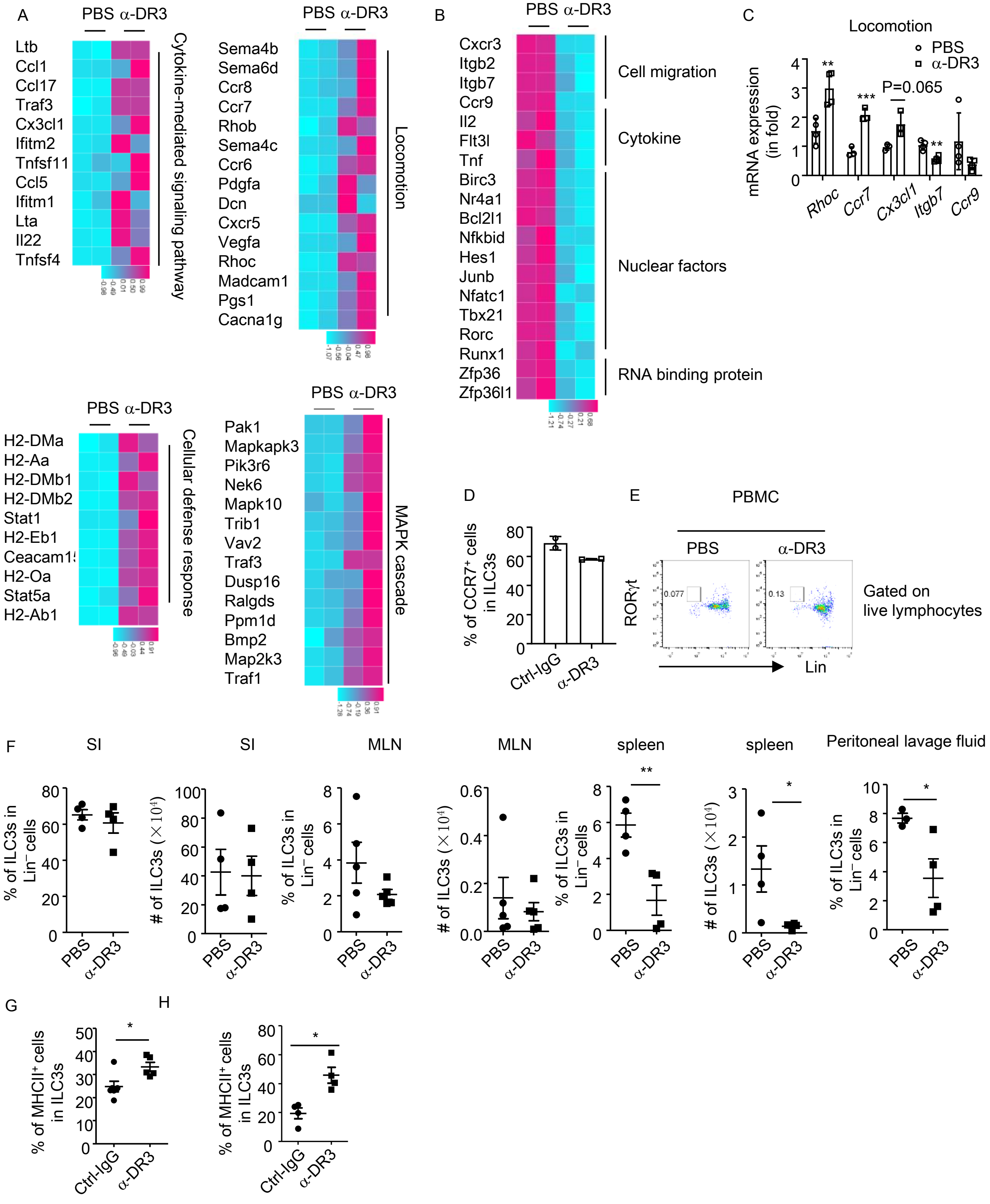

Supplementary Figure 5. α-DR3 regulates gene expression relevant to locomotion and promotes MHCII expression in ILC3s.

(A-C) *Rag1*$^{-/-}$*Rorc*$^{gfp/+}$ mice were treated with 1ug α-DR3 once and large intestinal LPLs were isolated 3 days later. (A and B) Duplicates mRNA of FACS purified ILC3s (Lin^{-}GFP^{+} cells) was exacted and subjected to genome-wide analysis (RNA-seq). Representative list of upregulated genes (A) or downregulated genes (B) categorized by gene ontology in α-DR3 treated mice were shown with Z-normalized heatmap. (C) Relative expression of indicated genes in ILC3s (Lin^{-}GFP^{+} cells) was analyzed by real-time RT-PCR and normalized to PBS group. (D-H) Wild-type mice (G) or *Rag1*$^{-/-}$ mice (D-F and H) were treated with PBS, Hamster IgG (Ctrl-IgG) or α-DR3 antibody and large intestinal LPLs were isolated for analysis 4 days later. (D) Percentages of CCR7 expression in ILC3s (Lin^{-}RORγt^{+}) was analyzed by flow cytometry and shown. (E and F) Small intestinal LPLs, lymphocytes from peripheral blood (PBMC), mesenteric lymph nodes (MLN), spleen and peritoneal lavage fluid were isolated. (E) Expression of lineage markers (CD3, B220, CD11b and CD11c) and RORγt gated on live lymphocytes from PBMC was analyzed by flow cytometry. Percentages of ILC3s (Lin^{-}RORγt^{+}) gated on live lymphocytes were shown. (F) Percentages and numbers of ILC3s (Lin^{-}RORγt^{+}) from indicated groups were calculated and shown. (G and H) Percentages of MHCII expression in ILC3s (Lin^{-}RORγt^{+}) was analyzed by flow cytometry and shown. (E, F, G and H) Data are means ± SEM. (C) Statistical analyses were performed using paired t-test. (A-H) Data are representative of at least 2 independent experiments. Source data are provided as a Source Data File.

Supplementary Figure 6

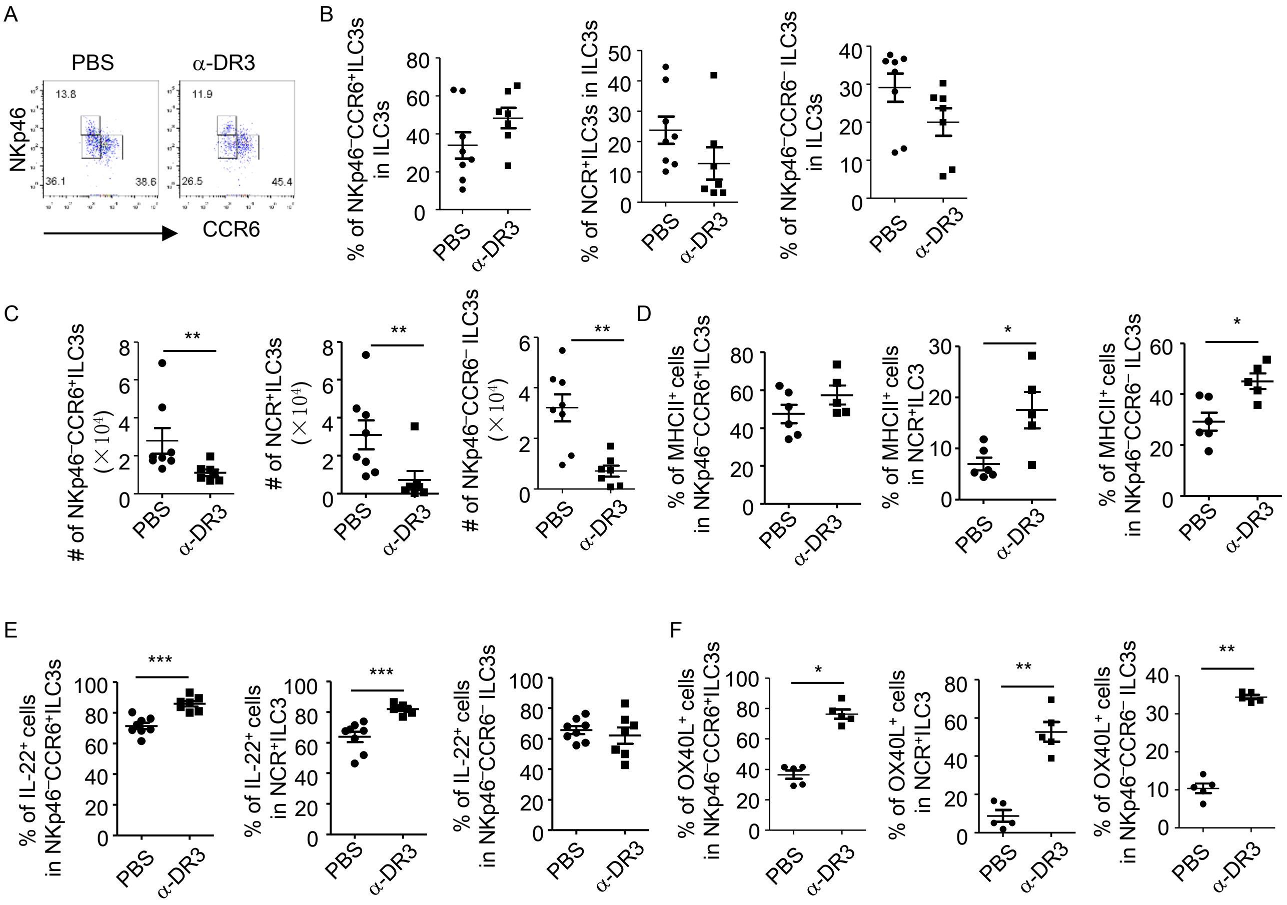

Supplementary Figure 6. α-DR3 treatment promotes the expression of OX40L, IL-22 and MHCII on subsets of ILC3s

Rag1$^{-/-}$ mice were treated with α-DR3 antibody (4C12) and large intestinal LPLs were isolated for analysis 4 days later. Expression of RORγt, NKp46, CCR6, MHCII, OX40L, IL-22 and Lineage markers (Lin, CD3, B220, CD11b, CD11c) was analyzed by flow cytometry. (A) Expression of NKp46 and CCR6 gated on ILC3s (Lin$^-$RORγt$^+$) was shown. (B)Percentages of CCR6$^+$NKp46$^-$ILC3s (Lin$^-$NKp46$^-$CCR6$^+$RORγt$^+$), NCR$^+$ILC3s (Lin$^-$CCR6$^-$NKp46$^+$RORγt$^+$) and CCR6$^-$NKp46$^-$ILC3s (Lin$^-$NKp46$^-$CCR6$^-$RORγt$^+$) gated on ILC3s (Lin$^-$RORγt$^+$) were shown. (C) Total numbers of CCR6$^+$NKp46$^-$ILC3s, NCR$^+$ILC3s and CCR6$^-$NKp46$^-$ILC3s from indicated groups were shown. (D-F) Expression of MHCII (D), IL-22 (E) and OX40L (F) gated on indicated subset of ILC3s was analyzed and shown. Data are means ± SEM (A-G) Data are representative of at least 2 independent experiments. Source data are provided as a Source Data File.

Supplementary Figure 7

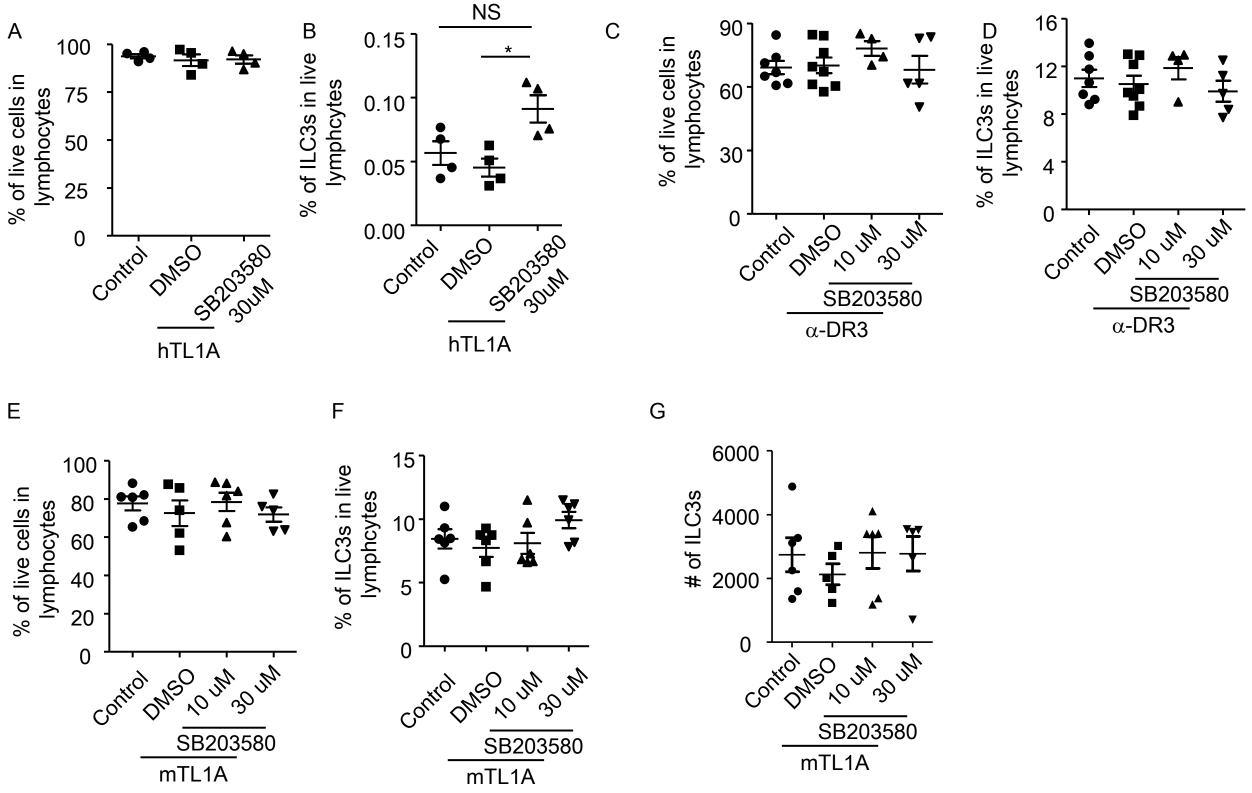

Supplementary Figure 7. SB203580 has no cytotoxic effect on ILC3s *in vitro*

(A and B) Human tonsil lymphocytes were treated with PBS or human recombinant TL1A (100ng/ml) in the presence or absence (DMSO) of inhibitor for p38 (SB203580) at 30uM for 18 hr. Brefeldin A was added for the last 2 hr before analysis. (C-G) Large intestinal LPLs were isolated from *Rag1*$^{-/-}$ mice. Cells were treated with PBS, α-DR3 (250ng/ml) or mouse TL1A (100ng/ml) in the presence or absence (DMSO) of inhibitor for p38 (SB203580) for 18 hr. Brefeldin A was added for the last 2 hr before analysis. (A, C and E) Percentages of live cells (dead cells-violet dye negative) gated on lymphocyte gate were analyzed by flow cytometry and shown. (B, D and F) Percentages of ILC3s (identified as Lin^{-}CD127^{+}CD117^{+} for B, as Lin^{-}RORγt^{+} for D and as CD45^{int}Thy1.2^{+}RORγt^{+} for F) gated on lymphocyte gate were analyzed by flow cytometry and shown. (G) Total numbers of ILC3s were calculated and shown when cells were harvested. Data are means ± SEM (A-G) Data are representative of at least 2 independent experiments. Source data are provided as a Source Data File.

Supplementary Figure 8

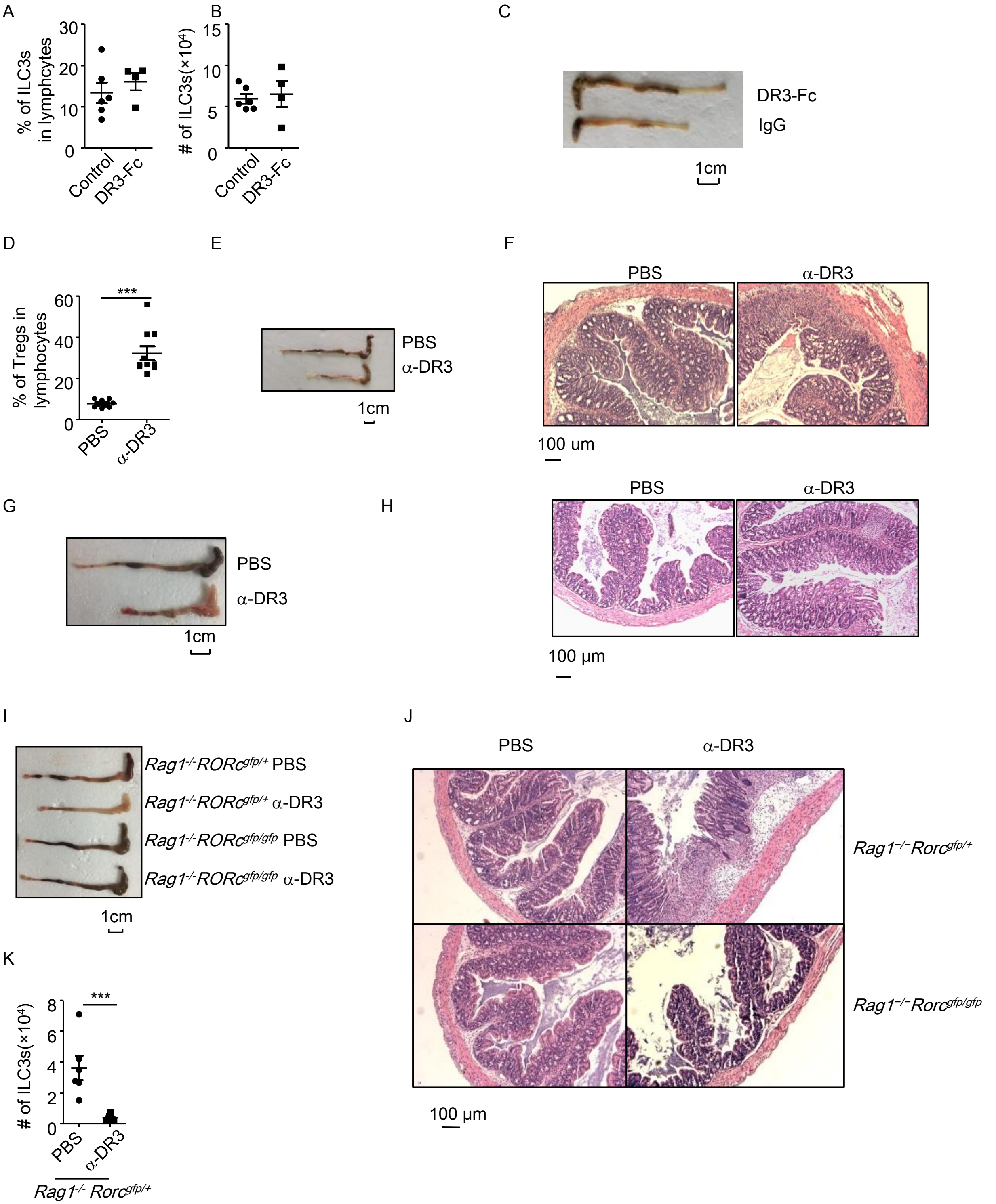

Supplementary Figure 8. ILC3s are important for exacerbation of DSS-induced innate colitis by α-DR3.

(A and B) Littermate *Rag1*$^{-/-}$ mice were treated with PBS (n=2), mouse IgG (n=4) or DR3-Fc (n=4) at 200ug per mouse daily for 3 days. Large intestinal LPLs were isolated and expression of RORγt and Lineage markers was analyzed by flow cytometry. Percentages of ILC3s (Lin^{-}RORγt^{+}) in lymphocytes and total numbers of ILC3s were shown. (C) Littermate *Rag1*$^{-/-}$ mice were fed with 3.25% DSS in drinking water for 4 days and sacrificed on day 7. Mice were treated with 200ug of mouse IgG or DR3-Fc in 200ul PBS from day 3-6. (D-K) Littermate wild-type (D-F), *Rag1*$^{-/-}$(G and H), *Rag1*$^{-/-}$*Rorc*$^{gfp/+}$ and *Rag1*$^{-/-}$*Rorc*$^{gfp/gfp}$ mice (I-K) were fed with 3.25% (for wild-type mice) or 3.5% (for mice of *Rag1*$^{-/-}$ background) in drinking water. (D-K) 2.5ug (for wild-type mice) or 1ug (for mice of *Rag1*$^{-/-}$ background) α-DR3 antibody or PBS was i.p. injected to mice on day -2, 0 and 2 post DSS treatment. Mice were sacrificed on analysis on day 4 post DSS treatment. (D and K) Large intestinal LPLs were isolated on day 4 post DSS treatment. Expression of foxp3, RORγt and Lin was analyzed by flow cytometry. (D) Percentages of Tregs (Lin^{+}Foxp3^{+}) in lymphocytes were shown. (K) Total numbers of ILC3s (Lin^{-}RORγt^{+}) were shown. (C, E, G and I) Representative pictures of large intestine from indicated groups were shown. (F, H and J) Paraffin-embedded colon sections were subjected to H&E staining and representative images in indicated groups were shown. Scale bar was 100um. (A, B, D and K) Data are means ± SEM. (A-K) Data are representative of at least two independent experiments. Source data are provided as a Source Data File.

Supplementary Table 1. List of antibodies used for flow cytometry

Clone numbers, fluorophore and manufacture of the antibodies used for flow cytometry were shown.

Antibody against	Clone or Kit	Fluorophore	Vendor	Reactivity	Dilution
B220	RA-6B2	FITC, PE-Cyanine7	Thermo Fisher Scientific	mouse	1:400
CCR7	4B12	PE-Cyanine7	Thermo Fisher Scientific	mouse	1:20
CD103	2E7	APC	Thermo Fisher Scientific	mouse	1:200
CD11b	M1/70	FITC, PE-Cyanine7	Thermo Fisher Scientific	mouse	1:400
CD11c	N418	FITC, PE-Cyanine7	Thermo Fisher Scientific	mouse	1:400
CD335	29A1.4	APC-eFluor® 780	Thermo Fisher Scientific	mouse	1:80
CD3e	145-2C11	FITC, PE-Cyanine7	Thermo Fisher Scientific	mouse	1:400
CD45	30-F11	PE-Cyanine7	Thermo Fisher Scientific	mouse	1:200
CD90.1	HIS51	PE	Thermo Fisher Scientific	mouse	1:200
CD90.2	30-H12	PE	Thermo Fisher Scientific	mouse	1:200
Cleaved caspase 3	CaspGLOWTM Red Active Caspase Staining Kit	NA	BioVision	mouse	1:100
DR3	4C12	PE	Biolegend	mouse	1:80
Foxp3	FJK-16s	FITC	Thermo Fisher Scientific	mouse	1:200
GATA3	L50-823	Alexa Fluor® 647	BD Biosciences	mouse	1:40
GM-CSF	MP1-22E9	FITC	Thermo Fisher Scientific	mouse	1:200
Ki-67	SolA15	PE	Thermo Fisher Scientific	mouse	1:200
Ly-6G	1A8-Ly6g	APC	Thermo Fisher Scientific	mouse	1:200
MHC II	M5/114.15.2	FITC	Thermo Fisher Scientific	mouse	1:200
NKp46	29A1.4	FITC, APC	Thermo Fisher Scientific	mouse	1:200
Phospho-p38	D3F9	NA	Cell Signaling Technology	mouse	1:200
RORγt	B2D	PE, APC	Thermo Fisher Scientific	mouse	1:200
Siglec-F	E50-2440	PE	BD Biosciences	mouse	1:200
CCR6	140706	BV421	BD Biosciences	mouse	1:20
CCR6	29-2L17	BV421	Biolegend	mouse	1:20
IL-22	Poly5164	PE	Biolegend	mouse	1:200
OX40L	RM134L	APC	Biolegend	mouse	1:200
CD11b	ICRF44	FITC	Biolegend	human	1:20
CD11c	3.9	FITC	Biolegend	human	1:20
CD127	A019D5	PE	Biolegend	human	1:40
CD14	M5E2	FITC	BioLegend	human	1:20
CD19	HIB19	FITC	Biolegend	human	1:50
CD3	UCHT1	FITC	BioLegend	human	1:50
CD3	SP34-2	APC-Cy™7	BD Biosciences	human	1:50
CD5	UCHT2	FITC	Thermo Fisher Scientific	human	1:20
c-Kit	104D2	PE/Cy7	Biolegend	human	1:20
FceRI	AER-37(CRA1)	FITC	Thermo Fisher Scientific	human	1:50
GM-CSF	BVD2-21C11	APC	Biolegend	human	1:10
Goat Anti-Rabbit IgG (H+L)	NA	Alexa Fluor 647	Jackson ImmunoResearch	rabbit	1:400

Supplementary Table 2. List of primers used for real-time RT-PCR

Sequences for primers used for real-time RT-PCR experiments were shown.

Gene	Forward	Reverse
Il22	5'-GACCAAACTCAGCAATCAGCTC-3'	5'-TACAGACGCAAGCATTTCTCAG-3'
Csf2ra	5'-ATCCTCTCGAGGCTGAGGAC-3'	5'-CGCGCACAGTAGGACTGC-3'
Csf2rb	5'-CGAGCTACTCTGGCAGAACT-3'	5'-TTTGTGGCTCTGTGCTTGGG-3'
Il23a	5'-CCAGTTCTGCTTGCAAAGG-3'	5'-GGTGATCCTCTGGCTGGA-3'
Il12a	5'-TCTCTATGGTCAGCGTTCCA-3'	5'-TTTCTCTGGCCGTCTTCAC-3'
Il12b	5'-TGGGAGTACCCTGACTCCTG-3'	5'-AGGAACGCACCTTTCTGGTT-3'
Ltb	5'-CAGCTGCGGATTCTACACCA-3'	5'-CATCCAAGCGCCTATGAGGT-3'
Tnfsf4	5'-CTGCCTGCAACTCTCTTCCT-3'	5'-TGACAACCGAATTGTTCTGC-3'
Zfp36	5'-CTACGAGAGCCTCCAGTCGAT-3'	5'-AGCCAAAGGTGCAAAACCAG-3'
Zfp36l1	5'- CAGATCCTAGTCCTTGCCCC-3'	5'-CGCTGGGAGTGCTGTAGTTG-3'
Rorgt	5'-CCGCTGAGAGGGCTTCAC-3'	5'-TGCAGGAGTAGGCCACATTACA-3'
Map2k3	5'-CTTGGAACCGTCGCGTCT-3'	5'-GGATTTTCCTTTGGTCTGAGGC-3'
Traf1	5'-TGCCAAGCTCATTTTAAAGCACA-3'	5'-TTGGCTACCCTATGTCACACG-3'
Tnfsf15	5'-TCCCATCCTCGCAGGACTTA-3'	5'-GCTGTGGTGAAGGCTCAGAT-3'
Rhoc	5'-CGGTGGAGCCCAAGTTTCA-3'	5'-GACAATGAGGAGGCAGGTCT-3'
Ccr7	5'-GAAACCCAGGAAAAACGTGCT-3'	5'-TTGAAGCACACCGACTCGTA-3'
Cx3cl1	5'-CTACTAGGAGCTGCGACACG-3'	5'-AAGCCACTGGGATTCGTGAG-3'
Itgb7	5'-GACCTTGGCCCCTAACTTGG-3'	5'-GATCCACTCCCTTCTCTTGGG-3'
Ccr9	5'-GTCTCAGTTCCCCTACAACTCC-3'	5'-CGGAATCTCTCGCCAACAAAA-3'
Actb	5'-CTTCTTTGCAGCTCCTTCGTT-3'	5'-AGGAGTCCTTCTGACCCATTC-3'
