# Supplementary material for: Activation of DR3 signaling causes loss of ILC3s and exacerbates intestinal inflammation
Source: Nat Commun. 2019 Jul 29;10:3371. doi: 10.1038/s41467-019-11304-8 (PMC6662828; doi:10.1038/s41467-019-11304-8)
Supplement: Supplementary file 3 — Description of Additional Supplementary Files [file 41467_2019_11304_MOESM3_ESM.pdf]

Description of Additional Supplementary Files

File name: Supplementary Data 1

Description: List of genes with significantly differential expression analyzed by RNA-seq. *Rag1*$^{-/-}$*Rorc*$^{gfp/+}$ mice were treated with PBS or 1ug of [illegible]-DR3 once and large intestinal LPLs were isolated 3 days later. Duplicates mRNA of FACS purified ILC3s (Lin^{-}GFP^{+}) cells was exacted and subjected to genome-wide analysis (RNA-seq). FPKM value in all detected genes in duplicate samples, genes with significant change in expression and genes with the expression significantly up or down for more than 1.5 fold were shown.
