# Supplementary material for: Activation of DR3 signaling causes loss of ILC3s and exacerbates intestinal inflammation
Source: Nat Commun. 2019 Jul 29;10:3371. doi: 10.1038/s41467-019-11304-8 (PMC6662828; doi:10.1038/s41467-019-11304-8)
Supplement: Supplementary file 5 — Reporting Summary [file 41467_2019_11304_MOESM5_ESM.pdf]

nature research

Corresponding author(s): Ju Qiu

Reporting Summary

Nature Research wishes to improve the reproducibility of the work that we publish. This form provides structure for consistency and transparency in reporting. For further information on Nature Research policies, see Authors & Referees and the Editorial Policy Checklist.

Statistical parameters

When statistical analyses are reported, confirm that the following items are present in the relevant location (e.g. figure legend, table legend, main text, or Methods section).

n/a	Confirmed	
☐	☒	The exact sample size (n) for each experimental group/condition, given as a discrete number and unit of measurement
☐	☒	An indication of whether measurements were taken from distinct samples or whether the same sample was measured repeatedly
☐	☒	The statistical test(s) used AND whether they are one- or two-sided<br>*Only common tests should be described solely by name; describe more complex techniques in the Methods section.*
☒	☐	A description of all covariates tested
☐	☒	A description of any assumptions or corrections, such as tests of normality and adjustment for multiple comparisons
☐	☒	A full description of the statistics including central tendency (e.g. means) or other basic estimates (e.g. regression coefficient) AND variation (e.g. standard deviation) or associated estimates of uncertainty (e.g. confidence intervals)
☐	☒	For null hypothesis testing, the test statistic (e.g. F, t, r) with confidence intervals, effect sizes, degrees of freedom and P value noted<br>*Give P values as exact values whenever suitable.*
☒	☐	For Bayesian analysis, information on the choice of priors and Markov chain Monte Carlo settings
☒	☐	For hierarchical and complex designs, identification of the appropriate level for tests and full reporting of outcomes
☒	☐	Estimates of effect sizes (e.g. Cohen's d, Pearson's r), indicating how they were calculated
☐	☒	Clearly defined error bars<br>*State explicitly what error bars represent (e.g. SD, SE, CI)*

Our web collection on statistics for biologists may be useful.

Software and code

Policy information about availability of computer code

Data collection	Raw data were analyzed by FlowJo V7 and V10 for flow cytometric data, by ImageJ for immunofluorescence, by HiSeq 2000 System for RNA-seq data.
Data analysis	Statistical analyses were performed with Graphpad Prism 5. For RNA-seq data, reads were mapped to Mouse Genome Assembly GRCm38.p5 by STAR v2.5. Gene and isoform expression quantification was called by RSEM v1.2 with default parameters on GENCODE mouse M16 gene annotation file. Differential expression analysis was performed by Bioconductor package edgeR v3.18.1. Heatmap was generated with software HemI 1.0.

For manuscripts utilizing custom algorithms or software that are central to the research but not yet described in published literature, software must be made available to editors/reviewers upon request. We strongly encourage code deposition in a community repository (e.g. GitHub). See the Nature Research guidelines for submitting code & software for further information.

Data

Policy information about availability of data

All manuscripts must include a data availability statement. This statement should provide the following information, where applicable:

- Accession codes, unique identifiers, or web links for publicly available datasets
- A list of figures that have associated raw data
- A description of any restrictions on data availability

Source data are provided as a Source Data File. Other datasets generated during and/or analysed during the current study are available from the corresponding author or first authors on reasonable request.

Field-specific reporting

Please select the best fit for your research. If you are not sure, read the appropriate sections before making your selection.

☒ Life sciences ☐ Behavioural & social sciences ☐ Ecological, evolutionary & environmental sciences

For a reference copy of the document with all sections, see nature.com/authors/policies/ReportingSummary-flat.pdf

Life sciences study design

All studies must disclose on these points even when the disclosure is negative.

Sample size	For all experiments in this study, at least 3 samples per group were used. For most experiments, 5-12 mice were included in each group. For experiments using small sample sizes, littermate mice were used and the data were from at least two independent experiments.
Data exclusions	No data were excluded in this study.
Replication	Data were pooled from at least two independent experiments. Consistent difference (or no difference) was observed, as indicated by the average value of each group, from at least two independent experiments. And statistical analyses were further performed based on the pooled data.
Randomization	Littermate mice were randomly grouped into control and treatment groups for all experiments in this study.
Blinding	Data were collected by the same person carrying out the expriments. Two major authors worked individually and collaboratively on the project. Therefore, the data were not collected in a blinded manner. However, histological scoring has been performed double-blindedly.

Behavioural & social sciences study design

All studies must disclose on these points even when the disclosure is negative.

Study description	*Briefly describe the study type including whether data are quantitative, qualitative, or mixed-methods (e.g. qualitative cross-sectional, quantitative experimental, mixed-methods case study).*
Research sample	*State the research sample (e.g. Harvard university undergraduates, villagers in rural India) and provide relevant demographic information (e.g. age, sex) and indicate whether the sample is representative. Provide a rationale for the study sample chosen. For studies involving existing datasets, please describe the dataset and source.*
Sampling strategy	*Describe the sampling procedure (e.g. random, snowball, stratified, convenience). Describe the statistical methods that were used to predetermine sample size OR if no sample-size calculation was performed, describe how sample sizes were chosen and provide a rationale for why these sample sizes are sufficient. For qualitative data, please indicate whether data saturation was considered, and what criteria were used to decide that no further sampling was needed.*
Data collection	*Provide details about the data collection procedure, including the instruments or devices used to record the data (e.g. pen and paper, computer, eye tracker, video or audio equipment) whether anyone was present besides the participant(s) and the researcher, and whether the researcher was blind to experimental condition and/or the study hypothesis during data collection.*
Timing	*Indicate the start and stop dates of data collection. If there is a gap between collection periods, state the dates for each sample cohort.*
Data exclusions	*If no data were excluded from the analyses, state so OR if data were excluded, provide the exact number of exclusions and the rationale behind them, indicating whether exclusion criteria were pre-established.*
Non-participation	*State how many participants dropped out/declined participation and the reason(s) given OR provide response rate OR state that no participants dropped out/declined participation.*

Randomization	*If participants were not allocated into experimental groups, state so OR describe how participants were allocated to groups, and if allocation was not random, describe how covariates were controlled.*

Ecological, evolutionary & environmental sciences study design

All studies must disclose on these points even when the disclosure is negative.

Study description	*Briefly describe the study. For quantitative data include treatment factors and interactions, design structure (e.g. factorial, nested, hierarchical), nature and number of experimental units and replicates.*
Research sample	*Describe the research sample (e.g. a group of tagged Passer domesticus, all Stenocereus thurberi within Organ Pipe Cactus National Monument), and provide a rationale for the sample choice. When relevant, describe the organism taxa, source, sex, age range and any manipulations. State what population the sample is meant to represent when applicable. For studies involving existing datasets, describe the data and its source.*
Sampling strategy	*Note the sampling procedure. Describe the statistical methods that were used to predetermine sample size OR if no sample-size calculation was performed, describe how sample sizes were chosen and provide a rationale for why these sample sizes are sufficient.*
Data collection	*Describe the data collection procedure, including who recorded the data and how.*
Timing and spatial scale	*Indicate the start and stop dates of data collection, noting the frequency and periodicity of sampling and providing a rationale for these choices. If there is a gap between collection periods, state the dates for each sample cohort. Specify the spatial scale from which the data are taken*
Data exclusions	*If no data were excluded from the analyses, state so OR if data were excluded, describe the exclusions and the rationale behind them, indicating whether exclusion criteria were pre-established.*
Reproducibility	*Describe the measures taken to verify the reproducibility of experimental findings. For each experiment, note whether any attempts to repeat the experiment failed OR state that all attempts to repeat the experiment were successful.*
Randomization	*Describe how samples/organisms/participants were allocated into groups. If allocation was not random, describe how covariates were controlled. If this is not relevant to your study, explain why.*
Blinding	*Describe the extent of blinding used during data acquisition and analysis. If blinding was not possible, describe why OR explain why blinding was not relevant to your study.*

Did the study involve field work? ☐ Yes ☒ No

Field work, collection and transport

Field conditions	*Describe the study conditions for field work, providing relevant parameters (e.g. temperature, rainfall).*
Location	*State the location of the sampling or experiment, providing relevant parameters (e.g. latitude and longitude, elevation, water depth).*
Access and import/export	*Describe the efforts you have made to access habitats and to collect and import/export your samples in a responsible manner and in compliance with local, national and international laws, noting any permits that were obtained (give the name of the issuing authority, the date of issue, and any identifying information).*
Disturbance	*Describe any disturbance caused by the study and how it was minimized.*

Reporting for specific materials, systems and methods

Materials & experimental systems

n/a	Involved in the study	
☐	☒	Unique biological materials
☐	☒	Antibodies
☐	☐	Eukaryotic cell lines
☐	☐	Palaeontology
☐	☒	Animals and other organisms
☐	☒	Human research participants

Methods

n/a	Involved in the study	
☐	☐	ChIP-seq
☐	☒	Flow cytometry
☐	☐	MRI-based neuroimaging

Unique biological materials

Policy information about availability of materials

Obtaining unique materials	In this study, the DR3-Fc protein was available from Biointron Biological Inc. Detailed cloning methods for plasmids that are used for hydrodynamic injection will be provided upon request. Other materials were commercially available.

Antibodies

Antibodies used	a-CD40 (FGK4.5), a-p40 (C17.8,), and a-p75 (R2-9A5) were from Bioxcel. a-p19 (G23-8) was from Thermo Fisher Scientific. a-DR3 (4C12) was from Biolegend. List of antibodies used for flow cytometry was provided as Table S3 in the manuscript. a-GATA3 Alexa Fluor® 647(L50-823), a-Siglec-F PE(E50-2440), a-human CD3 APC-Cy™7(SP34-2) were from BD Biosciences. a-mouse DR3 (TNFRSF25) PE(4C12), a-mouse CD335 (NKp46) APC(29A1.4), a-human CD11c FITC(3.9), a-human CD11b FITC(ICRF44), a-human CD19 FITC(HIB19), a-human CD3 FITC(UCHT1), a-human CD14 FITC(M5E2), a-human CD127 (IL-7Rα) PE(A019D5), a-human CD117 (c-kit) PE/Cy7(104D2), a-human GM-CSF APC(BVD2-21C11) were from Biolegend. a-mouse CD11b PE-Cyanine7(M1/70), a-mouse CD11c PE-Cyanine7(N418), a-mouse CD3e PE-Cyanine7(145-2C11), a-human/mouse CD45R (B220) PE-Cyanine7(RA3-6B2), a-mouse CD127 FITC(A7R34), a-mouse CD335 (NKp46) APC-eFluor® 780(29A1.4), a-mouse CD11b FITC(M1/70), a-mouse Ly-6G (Gr-1) APC(1A8-Ly6g), a-mouse CD103 (Integrin alpha E) APC(2E7), a-mouse MHC Class II (I-A/I-E) FITC(M5/114.15.2), a-mouse/rat CD90.1 (Thy-1.1) PE(HIS51), a-mouse GM-CSF FITC(MP1-22E9), a-mouse/rat Foxp3 FITC(FJK-16s), a-mouse CD335 (NKp46) FITC(29A1.4), a-mouse/rat Ki-67 PE-Cyanine7(SolA15), a-mouse CD197(CCR7) PE(4B12), a-human CD5 FITC(UCHT2), a-human FceRI FITC(AER-37 (CRA1)) were from Thermo Fisher Scientific. Phospho-p38 MAPK (Thr180/Tyr182) (D3F9) XP® Rabbit mAb was from Cell Signaling Technology. All the antibodies have been tested by the manufacture for application in stimulation, neutralization or flow cytometry.
Validation	Primary antibodies used in this study have been validated by the manufactures.

Eukaryotic cell lines

Policy information about cell lines

Cell line source(s)	*State the source of each cell line used.*
Authentication	*Describe the authentication procedures for each cell line used OR declare that none of the cell lines used were authenticated.*
Mycoplasma contamination	*Confirm that all cell lines tested negative for mycoplasma contamination OR describe the results of the testing for mycoplasma contamination OR declare that the cell lines were not tested for mycoplasma contamination.*
Commonly misidentified lines (See ICLAC register)	*Name any commonly misidentified cell lines used in the study and provide a rationale for their use.*

Palaeontology

Specimen provenance	*Provide provenance information for specimens and describe permits that were obtained for the work (including the name of the issuing authority, the date of issue, and any identifying information).*
Specimen deposition	*Indicate where the specimens have been deposited to permit free access by other researchers.*
Dating methods	*If new dates are provided, describe how they were obtained (e.g. collection, storage, sample pretreatment and measurement), where they were obtained (i.e. lab name), the calibration program and the protocol for quality assurance OR state that no new dates are provided.*

☐ Tick this box to confirm that the raw and calibrated dates are available in the paper or in Supplementary Information.

Animals and other organisms

Policy information about studies involving animals; ARRIVE guidelines recommended for reporting animal research

Laboratory animals	Wild-type or genetically engineered mice with the C57BL/6 background have been used in this study. Littermate mice were used for in vivo studies. Specifically, female litteramtes were used for DR3-Fc-treatment experiments. And both male and female mice have been used in experiments with a-DR3 injection.
Wild animals	The study didn't involve wild animals.
Field-collected samples	This study didn't involve samples collected from the feild.

Human research participants

Policy information about studies involving human research participants

Population characteristics	Tonsil sections from both female and male patients, with age ranging from 37 to 55.
Recruitment	Human tonsils were from patients subjected to tonsillectomies. Tonsil cells from both male and female with ages ranging from 37 to 55 were involved this study for only in vitro experiments. There is no self-selection bias in this study.

ChIP-seq

Data deposition

☐ Confirm that both raw and final processed data have been deposited in a public database such as GEO.

☐ Confirm that you have deposited or provided access to graph files (e.g. BED files) for the called peaks.

Data access links<br>*May remain private before publication.*	*For "Initial submission" or "Revised version" documents, provide reviewer access links. For your "Final submission" document, provide a link to the deposited data.*
Files in database submission	*Provide a list of all files available in the database submission.*
Genome browser session<br>(e.g. UCSC)	*Provide a link to an anonymized genome browser session for "Initial submission" and "Revised version" documents only, to enable peer review. Write "no longer applicable" for "Final submission" documents.*

Methodology

Replicates	*Describe the experimental replicates, specifying number, type and replicate agreement.*
Sequencing depth	*Describe the sequencing depth for each experiment, providing the total number of reads, uniquely mapped reads, length of reads and whether they were paired- or single-end.*
Antibodies	*Describe the antibodies used for the ChIP-seq experiments; as applicable, provide supplier name, catalog number, clone name, and lot number.*
Peak calling parameters	*Specify the command line program and parameters used for read mapping and peak calling, including the ChIP, control and index files used.*
Data quality	*Describe the methods used to ensure data quality in full detail, including how many peaks are at FDR 5% and above 5-fold enrichment.*
Software	*Describe the software used to collect and analyze the ChIP-seq data. For custom code that has been deposited into a community repository, provide accession details.*

Flow Cytometry

Plots

Confirm that:

☒ The axis labels state the marker and fluorochrome used (e.g. CD4-FITC).

☒ The axis scales are clearly visible. Include numbers along axes only for bottom left plot of group (a 'group' is an analysis of identical markers).

☒ All plots are contour plots with outliers or pseudocolor plots.

☒ A numerical value for number of cells or percentage (with statistics) is provided.

Methodology

Sample preparation	1. The isolation of intestinal lamina propria cells was done as below. Briefly, large intestines were dissected, fat tissues and peyer's patches were removed. Intestines were cut open longitudinally and washed in PBS. Intestines were then cut into 3cm-long pieces, washed and shaken in PBS containing 1 mM DTT for 10 min at RT. Intestines were incubated with shaking in PBS containing 30 mM EDTA and 10 mM HEPES at 37°C for 10 min for two cycles. Supernatant from the first round of EDTA was saved as intestinal epithelial cells. The tissues were then digested in RPMI1640 medium (Thermo Fisher Scientific) containing DNase I (150ug/ml, Sigma) and collagenase VIII (200U/ml, Sigma) at 37°C in 5% CO2 incubator for 1.5 hr. The digested tissues were homogenized by vigorous shaking and passed through 100um cell strainer. Mononuclear cells were then harvested from the interphase of an 80% and 40% Percoll gradient after a spin at 2500 rpm for 20 min at room temperature.<br>2. Human tonsil tissues were cut into 3-10 mm fragments and mechanically disrupted using the plunger end of a plastic syringe. Cell suspensions were filtered through a 70um cell strainer, and mononuclear cells were isolated with LymphoprepTM (Axis Shield).<br>3. Cell suspension preparation from peritoneal lavage fluid, spleen or mesenteric lymph nodes<br>Peritoneal lavage cells were isolated by flushing the peritoneal cavity with 10ml PBS. Cell suspensions were prepared from spleen, and mesenteric lymph nodes by gentle mechanical disruption and passed through a 50 um nylon mesh. Blood was

	collected by the heart punctures. Mononuclear cells from the blood were isolated from interface of Ficoll-Paque density gradient centrifugation at 2500 rpm for 20 min at room temperature.
Instrument	Moflo Astrios and Gallios flow cytometer (Beckman).
Software	Flowjo V10 and V7 software.
Cell population abundance	About 0.2 million ILC3s (Lin–GFP+) were got from the large intestinal lamina propria lymphocytes from 3-6 mice. About 0.12 million CD11blowCD11c+ cells, 0.1 million CD11b+CD11c+CD103+ cells, 0.17 million CD11b+CD11c+CD103- cells and 0.19 million eosinophils were got from Rag1-/- mice. Cells were sorted with Moflow Astrios (Beckman) and purity of cells were examined with Gallios (Beckman) flow cytometry. The purity was more than 95%.
Gating strategy	For analysis of ILCs, lymphocytes were firstly gated based on FSC/SSC. For analysis of myeloid cell populations, lymphocytes and large cells were all gated based on FSC/SSC. Positive and negative cells were identified based on clear boudaries between the two populations. For markers with no clear boudaries between positive and negative populatioins, FMO was used as a control.

☒ Tick this box to confirm that a figure exemplifying the gating strategy is provided in the Supplementary Information.

Magnetic resonance imaging

Experimental design

Design type	*Indicate task or resting state; event-related or block design.*
Design specifications	*Specify the number of blocks, trials or experimental units per session and/or subject, and specify the length of each trial or block (if trials are blocked) and interval between trials.*
Behavioral performance measures	*State number and/or type of variables recorded (e.g. correct button press, response time) and what statistics were used to establish that the subjects were performing the task as expected (e.g. mean, range, and/or standard deviation across subjects).*

Acquisition

Imaging type(s)	*Specify: functional, structural, diffusion, perfusion.*
Field strength	*Specify in Tesla*
Sequence & imaging parameters	*Specify the pulse sequence type (gradient echo, spin echo, etc.), imaging type (EPI, spiral, etc.), field of view, matrix size, slice thickness, orientation and TE/TR/flip angle.*
Area of acquisition	*State whether a whole brain scan was used OR define the area of acquisition, describing how the region was determined.*

Diffusion MRI ☐ Used ☐ Not used

Preprocessing

Preprocessing software	*Provide detail on software version and revision number and on specific parameters (model/functions, brain extraction, segmentation, smoothing kernel size, etc.).*
Normalization	*If data were normalized/standardized, describe the approach(es): specify linear or non-linear and define image types used for transformation OR indicate that data were not normalized and explain rationale for lack of normalization.*
Normalization template	*Describe the template used for normalization/transformation, specifying subject space or group standardized space (e.g. original Talairach, MNI305, ICBM152) OR indicate that the data were not normalized.*
Noise and artifact removal	*Describe your procedure(s) for artifact and structured noise removal, specifying motion parameters, tissue signals and physiological signals (heart rate, respiration).*
Volume censoring	*Define your software and/or method and criteria for volume censoring, and state the extent of such censoring.*

Statistical modeling & inference

Model type and settings	*Specify type (mass univariate, multivariate, RSA, predictive, etc.) and describe essential details of the model at the first and second levels (e.g. fixed, random or mixed effects; drift or auto-correlation).*
Effect(s) tested	*Define precise effect in terms of the task or stimulus conditions instead of psychological concepts and indicate whether ANOVA or factorial designs were used.*

Specify type of analysis: ☐ Whole brain ☐ ROI-based ☐ Both

Statistic type for inference (See Eklund et al. 2016)	*Specify voxel-wise or cluster-wise and report all relevant parameters for cluster-wise methods.*

Correction

Describe the type of correction and how it is obtained for multiple comparisons (e.g. FWE, FDR, permutation or Monte Carlo).

Models & analysis

n/a	Involved in the study
☐	☐ Functional and/or effective connectivity
☐	☐ Graph analysis
☐	☐ Multivariate modeling or predictive analysis

Functional and/or effective connectivity

Report the measures of dependence used and the model details (e.g. Pearson correlation, partial correlation, mutual information).

Graph analysis

Report the dependent variable and connectivity measure, specifying weighted graph or binarized graph, subject- or group-level, and the global and/or node summaries used (e.g. clustering coefficient, efficiency, etc.).

Multivariate modeling and predictive analysis

Specify independent variables, features extraction and dimension reduction, model, training and evaluation metrics.
